# Meeting of the Fulton County Medical Society

**Published:** 1915-05

**Authors:** R. R. Daly


### FULTON COUNTY MEDICAL SOCIETY.

### Regular Meeting May 6th, 1915.

By R. R. Daly, M. D.

Dr. T. C. Davison presented a case of gunshot wound of the spine, which is described elsewhere.

Dr. J. L. Campbell presented a case of paralysis of the left arm and forearm due to an injury to the shoulder, that was reflected to the brachial plexus.

The woman, colored, 45 years old, was chopping wood last December when a large piece struck her face and right shoulder. She was unconscious for a time and then showed paralysis of parts of the upper extremity. She was sent here last week with the diagnosis of paralysis of the musculo spiral nerve. An examination showed, however, that there was paralysis of every part of the arm and forearm that was supplied by the brachial plexus. The areas where there remained some sensation were supplied by the thoracic nerves or filiaments from the cervical plexus that did not touch the brachial.

X-Ray plates were negative as to injury. Muscles reacted equally to both poles in the musculo spiral region, showing no degeneration at present.

He is at a loss to know just what happened. The brachial plexus is tough and not easily torn and there is nothing in the injury to cut it, yet it is evidently interfered with.

Dr. Davison said that in Murphy's clinic in Chicago last October, he saw a very similar case. Murphy made a dissection and found that the clavicle had been struck downward by the injury enough to tear the brachial plexus beneath it, yet there was no fracture of the bone.

Dr. Baxter Moore asked if there had been manipulation of the shoulder joint under anaesthesia to be sure there has not been dislocation downward and pressure upon the plexus. The woman is very large and the points and landmarks are not easily felt, but it seems to him as if there was some dislocation farther than the normal one inch glenoid relaxation when the muscles are paralyzed.

Dr. H. I. Battey asked if there were any evidence of a cervicle rib that might have caused the trouble.

Campbell said there was no such rib. The point that Moore made was a good one and he would use an anesthetic.

Dr. G. C. Mizell presented a case simulating Pellagra.

The patient, Mr. G. B., referred by Dr. J. C. Wall, of Eastman, Ga., gave the following history: Age 35; married; family history unimportant; past history: Pneumonia when a child; jaundice at 25 years of age. Sick headache about once a month for past seven years, but more frequently recently. Present history: Began as a sick headache one month ago and has suffered with headache almost continually to the present time. Has lost 19 pounds; present weight, 121 pounds.

Eats general diet (native meats and eggs). Has bad taste in mouth and belches a great deal. He is often nauseated and vomits when headache is severe. He is dizzy and tends to go to one side. He is constipated, nervous and sleeps irregularly. He sweats very little at any time and has always had a more or less dry skin. The skin on back of hands has always tanned and thickened somewhat in summer. This has been more pronounced this year and is due to having his hands much in creosote and carbolic acid solution.

Examination shows: Pulse 86; blood pressure 110. Knee jerk slightly exaggerated.

Physical examination is negative except gastroptosis and sensitive over sigmoid. Urin SpGr. 1008, no albumen or sugar. Acid. No indican.

Ewald test breakfast gives, three ounces of residue, no hydrochloric acid.

Epidermis on hands is thickened, glazed and pigmented. It has the appearance of the moderate dermatitis of pellagra just after the erythema has faded. This condition of the skin in connection with anacidity suggests pellagra, but the history shows plainly that the dermatitis is clearly an occupational dermatitis and in regard to the gastric condition Mizell pointed out the absence of "pyrosis" or burning in the stomach or esophagus. He states that in all cases of pellagra that had affected the stomach to such a degree, i. e., anacidity, a history of burning in the esophagus or stomach could be obtained.

He states that there may be burning in non-pellagrous anacidity or subcidity from fermentation so that when it is present it does not always mean pellagra; but when it is absent in either case pellagra may be excluded.

Another point of interest pointed out in this case is the absence of intestinal autointoxication which is so common in anacidity and which may be accounted for by the fact that he is eating no cold storage meats and eggs.

Dr. A. W. Fowler read a paper on "The Small Fibrous Prostate." It is published elsewhere in the Journal.

Dr. W. F. Shallenberger read a paper "Urethritis in Women and a New Method of Diagnosis and Treatment in Obscure Urethral Pain."

His special point was the blocking off of pain by injection of cocaine or urea hydrochloride so that an examination could be made easily. In addition it seems clear that if the distress the patient suffers is relieved even for a short time by anesthetizing this area the cause of the pain must be in the area. He urged the necessity for a routine examination of the urethra whenever there were any genito-urinary pains. Many diseases have their origin in the urethra and if it is not examined, many etiologic factors are overlooked.

Dr. E. G. Ballenger read a paper "Further Report on Approved Method of Drainage after Cystotomy and Suprapubic Prostatectomy." He demonstrated his apparatus and described it in detail. It provides for intermittent or constant drainage or irrigation as the operator desires and at the same time stimulates healing by slight induced hyperaemia at the wound.

Dr. W. B. Emery said in discussing these papers that they all contained matter that was of great interest to the general practitioner and was of practical benefit to him in their suggestions. Ballenger's apparatus is excellent and meets a need Emery has felt for a long time. Others have leaked and the condition of the wound and dressings was no better because of them. Many people think that urine is good for the skin and will say so in the hospitals when one is trying to protect them from it, but it doesn't prove so around a wound. Shallenberger's insistence upon studying the urethra in all cases is well founded. Fowler's ideas in regard to the small prostate are important and the physician should keep them in mind whenever he has to deal with complaint of residual urine. It is true that a retrograde cystoscope is necessary to make the diagnosis and not everyone has this, but access to them can be had when they are demanded.

Dr. E. P. Merritt said that the matter of small prostate had never been made prominent in the text books and it was timely on that account alone. In addition it solved many problems that have long perplexed us.

Dr. E. C. Davis said that Shallenberger's paper attracts attention to an important area and indicates that cures are readily made. His own cases had not been so fortunate. Possibly it was because of the reinfection that came from the primal source and that the husband was not treated at the same time.

Dr. E. C. Cartilege said that he had what appeared at first sight to be one of Fowler's cases and he could not introduce a large sound. He noted that the meatus was small and cut it. This allowed a 32 sound to pass. The man complained of impotence and wanted sounds passed for stimulating purposes. He had diabetes and Cartilege had noted three other cases of diabetes with this defect. He asked if others had had similar experience.

Dr. M. L. Boyd said that his practice in suprapubic cases had been to drain the urine at regular intervals rather than to irrigate because he had no good apparatus. If Ballenger's method is practical it will be of great help. He prefers the perineal operative route even in the small enlargements of the prostate because he believes the parts may be more easily seen and besides, the ejaculatory ducts are safer.

Fowler said that the mucus membrane in the female urethra has three distinct layers: the flat, the columnar and the cuboidal in cell formation. These can be shown in the microscope and thus the intensity of the inflammation can be gauged by the cells thrown off. In the deep layer the inflammation is not likely to respond to Shallenberger's treatment of silver nitrate. Notwithstanding adverse statements, these different epithelial cells can be differentiated and pathologists here and abroad are teaching it today. He thinks that the tubes as shown in Ballenger's apparatus are too small and that clots might clog them. The irrigations might well be quite warm from 110 to 115 Fahr. The active hyperaemia the cup produces would lead to passive hyperaemia and this to passive congestion which would interfere with healing. He uses long tubes an inch in diameter to begin with, himself.

Shallenberger said that his cases were practically all not open to reinfection as Davis suggested and that almost none were specific. If Skene's glands were infected and there appeared to be suburethral abscesses, he slit them and drained. He had no trouble on this score.

Ballenger said that the tube he showed was smaller than the one he uses. Any size tube may be selected and can be placed either directly in the bladder or in the suction part of the apparatus. In practice the active hyperaemia he induces has none but good results and the wound heals nicely.

The condition of small fibrous prostate should be remembered whenever there was obstruction to the urinary flow. It is the ring rather than the gland that must be removed or destroyed as Fowler says. There are various avenues of approach and methods of treating. Chetwood makes a perineal incision and cauterizes deeply destroying the stricture. It may also be cut in several places.

### REGULAR MEETING MAY 20, 1915.

Dr. G. D. Ayer presented two cases in which he had operated successfully for Mastoid conditions.

One was a man of 35 who had had pain in ear and behind it for four months. He was unable to sleep the last two weeks before operation and he had temperature 101-102, together with swelling in the neck. Operation disclosed non-infected sinus thrombosis and it became necessary to do jugular ligation. In 20 days he was well and the hearing is good. Incidentally he had diabetes and the sugar is somewhat less in percentage than it was.

The other case was a girl of twelve who had pain in ear and mastoid swelling in the neck and high temperature, 104 to 105. A diagnosis of sinus thrombosis was made. Operation displayed epidural abscess with sinus intact. She made a good recovery except that the temperature persisted for many days. A culture showed that it was due to a streptoccus infection that eventually passed.

Dr. F. W. McRae said that the diabetic matter in the first case was important. He has operated upon many cases of Diabetes for other purposes and a large number of them improve when the strain is removed with the offending area operated upon.

Dr. R. M. Nelson said that he had a patient with 7% sugar whom he treated until it was reduced to 3% where he felt safe in operating. He then removed a subperiostal temporal abscess and the necrosed bone around it. The wound looked as if a mastoid had been done before. The patient made a good recovery from the abscess and the per cent of sugar was practically negligible.

The regular program of the evening was taken up. It was a surgical meeting and comprised four papers as follows:

1. Preliminary Operative Treatment, W. F. Westmoreland.

2. Surgical Technique and Anaesthesia. Remarks on Surgical Ideals and End Results, F. W. McRae.

3. The Time Element in Operations, W. P. Nicolson.

4. Post-Operative Treatment, E. G. Jones.

Dr. Westmoreland said that we have all realized that terminal results in surgery are not what they should be and that there have been almost no improvements in this regard during the last decade so far as change of methods is concerned. A close analysis of the end results leads us to consider several factors in our work and the Preliminary Treatment is not the least by far.

Leaving aside emergencies, we are often likely to operate too soon. A man from out of town brings a patient whom he wants cared for at once so that he can return home and urges us to immediate work. It is a temptation to oblige him just as it is to protect oneself from loss of time when one is called away to operate. But it isn't good surgical policy. The surgeon should take time to assure himself of the diagnosis by every means and the patient should have opportunity for rest and preparation before going on the table.

We have the general preparation and the local preparation of the field to consider.

The general preparation takes two forms. The health of the patient and his digestive and vegetative organs are to be noted. Elimination and rest are to be afforded and sufficient time must be given for this, preferably in the hospital.

In addition in the abdominal cases there is always likely to be autointoxication and some fermentation to be removed as far as possible before going to the table. Many of the postoperative adhesions are due to gas and intoxication that prevent rest coming to the patient immediately after operation. It is true that some intoxications are due to mechanical obstructions that must be removed but these are different cases.

In the local preparation of the patient, the important thing to consider in addition to the cleansing technique is the size of the field. The entire abdomen should be cleansed no matter what small area of it one thinks he needs. It induces better cleansing for one thing and gives room for other incisions if needed without delay.

Small incisions give bad results as well as too much work through one incision. Every incision should be large enough to admit the hand. Many return cases and adhesions are due to lack of this sort of preparation.

As to Diet, too little food is now given before operation. The liquid diet prescribed is likely to form gas and be in the way. A fairly generous diet when not obviously contraindicated is advisable and with this for several days before operating, the patient should be saturated with water. This will prevent dehydration of vomiting after returning to bed.

Catharsis as a routine is given too close to the operation. It should be administered two days before and be well out of the way. Besides, the patient should be allowed to rest and all nervous tension removed as far as possible two or three days prior to the work.

Dr. McRae spoke of the Surgical Technique, Anaesthesia, etc. It seems wise to go into details and take stock of even the least of things in our work. It is upon these, indeed, that many people differ as to degree of care and they thus affect the end results more than we realize. The end results mark success or lack of it and everything controlling them is of major importance. For example, two surgeons were doing about the same amount of work in four different hospitals. They noted 5% of infection in two of the hospitals where the instruments were cleansed and sterilized with alcohol and other chemicals. This disappeared when the instruments were boiled.

A systematized technique is necessary to real success in surgery. The hands should be cared for before and after operations and between times. No hand that has been bathed in pus is safe for two days, no matter what cleansing it receives. His method of caring for the hands is given with the comment that while this may seem very elementary and should be known by everyone attempting to do surgical work, it is often disregarded in some slight measure and the little carelessness results in infection.

He washes his hands first in soap and water and then adds benzine to the lather. This gets all the oil and dirt away from the hair follicles. Then soft sterile brush and soap and water all to the elbow. Then he uses the crystal carbonate of soda and chloride of lime, one to four, with water. This makes a nice cream that does not injure the hands or give off an offensive amount of free chlorine gas. It does sterilize the hands. Any other reagent used with the lime will give off too much chlorine and interfere with the good condition of the skin and bother the operator by the irritating fumes. After this he uses sterile water and dries on a sterile towel; 95% alcohol is applied to hands and wrists to make them dry. After this he puts on wet gloves. No powder is used because it interferes with sense of touch. The gloves should be taken out of a solution of cyanide of mercury 1 to 2000. Then if there should be any puncture of the gloves he is satisfied that no infection could come from hands. He has had his hands and those of his assistants examined after two hours hard work and they were sterile in the gloves. His infections have come only from sutures that passed through the patient's skin. This may be an argument for having sutures come from within outward.

Dr. Westmoreland's ideas of preparation are excellent, but he would extend them to the care of the patient's skin almost as he does his hands. The final touch of covering the entire field with iodine adds very greatly to safety. Then he wants the skin dry when he is at work.

He believes in absorbable sutures and uses them regularly unless otherwise particularly indicated. All trauma should be avoided. In this connection the Trendelenberg position and the packing off of areas are bad and cause shock and increased pulse rate as any good anaesthetist will say.

Anaesthesias should be standardized. We know what the defects of ether and chloroform are and why we want to get away from them when possible. Gas-ether is excellent. Crile's anoci association is invaluable whenever applicable. Then there are the other combinations of nitrous oxide gas and lastly the local anaesthetics. Something more definite should emerge from our experience regarding them and establish a standard.

Qualified anaesthetists are necessary and certainly no one not a doctor should be permitted to give anaesthetics. The training of nurses to do it is attended with the risk of their working with too limited knowledge.

Our surgical ideals should be kept in our mind's eye so as to know as far as in us lies what we are going to do; to know how the work should be done; to do it with the least possible trauma, exposure and loss of blood; to do all that is necessary and only what is necessary; to put yourself in the other fellow's place and show him that kind of consideration; to develop, cultivate and live up to one's surgical conscience.

Then as end results we will merit and win the confidence, love and esteem of our patients. We will minimize our mortality and post-operative morbidity. We will deserve and in large measure receive the respect and confidence of our fellows.

Dr. W. P. Nicolson spoke of the Time Element in Operations. From the patient's standpoint it is evident that no man wants to be asleep two hours if the work can be done in one. He will seek the rapid operator. It should be clear in the outset that rapid work is different from hurried work and that the latter is quite as dangerous as any other defect in method. Every surgeon should analyze and study his own methods so as to conserve time because each five minutes on the table adds to the ill effects the patient must ultimately withstand.

As to what makes men of equal large experience vary in operative time it is hard to say. Personal equation is only another name for the difference, but there are men of slow habit who can make haste in emergencies; and habit and training have much to do with speed or lack of it. Among other things contributing to celerity of work is accuracy of preparation for it. Every instrument should be selected and put in place; every possible emergency should be foreseen so that when it arises, no time is wasted in discussion or vacilating thought. Anatomical knowledge and surgical principles should be so completely in hand that one uses them as he does the letters of the alphabet in reading—accurately and exactly without conscious effort. Moreover, the dissection in an operation is not that fine separation that one resorts to in the dissection room and it should take much less time. Blunt dissection by the fingers is best whenever possible.

Not every abdominal operation should be an exploratory one. The surgeon should have determined by the use in advance of every diagnostic aid what the condition is and then attend to that. Thus he still believes in small incisions. They can be enlarged if necessary. The patient should be protected from every avoidable touch.

Surgical "piddling" in pretty small and numerous stitches should be avoided. It takes much time. If three stitches will hold the parts in apposition, why use more? We have fashions in surgery just as we do in everything else, but we must not let them take valuable time.

Team work with assistants is of the utmost importance and saves much time.

Dr. E. G. Jones spoke on Post-Operative Treatment.

He confined himself to such cases as would be likely to get well and left aside the large problems of great shock, secondary hemorrhage and obstruction of the bowels.

During the last ten years, the comfort of the patient has been added to by trained anaesthetists and the care they are able to give both before and during the operation. It is recognized that the patient will vomit when ether is used because of the ether itself; and various antiemetics are no longer given prior to the work. The eliminating organs, skin, kidneys, lungs and alimentary canal, all do their part in assisting the patient to recover, but he is intoxicated by the ether and must suffer its effects in the way of nausea just as he has enjoyed them in the way of unconsciousness. Lavage of the stomach is sometimes of value in persistent vomiting, but not until twenty-four hours after leaving the table. When given immediately it is not only not productive of good results, but it adds to the already great stress of the patient. The stomach doesn't or shouldn't contain anything at that time anyhow.

When vomiting has continued more than 24 hours, one must think of the conditions that may cause it. Acute nephritis, acute dilatation of the stomach, obstruction of the bowels or the opium that may have been given prior to the operation, and any other thing the condition of the patient may suggest must be considered.

Thirst after operation is troublesome, but there is no danger of dehydration if water is denied until vomiting has passed. Rectal injection will care for it if needed. The nurses say that there seems to be less nausea following the use of small amounts of hot water when given early. Morphine and atropine should not be given just for the thirst.

Pain after operation should be thought of in advance. The ordinary wound carefully handled and sutured will not be painful as a rule. But sometimes dressings are too tight or cling and pull in the drying secretions and at others a suture is binding. These should be relieved and the patient allowed all the comfort he can get. In the packing for hemorrhage care should be taken not to get things too tight. In any event, it is well to change all dressings in five or six days and let the patient have the comfort of feeling clean and tidy. Remove suture with sharp scissors.

Food after operation should be withheld for 12 hours, then perhaps egg albumen or grape juice may be given. The prepared and marketed foods and stimulants are aside from alcohol of no more significance than so much urine.

Dr. G. P. Huguley opened the discussion of the talks. He said that he had been a surgical case, himself, and knew the post-operative state was very uncomfortable. He uses opiates freely in his cases whenever indicated and has never known of habit formation. Not much is really needed. The anoci association prevents much of the nausea; proctoclysis and injection of soda bicarb and glucose does away with thirst; pituitrin has put aside the need for catheter and enemas. When one has good peristalsis, he does not have adhesions later on.

Dr. H. I. Battey said that patients are led to avoid operation often because of the distress attending them and the likelihood of having to return for secondary work. If all the precautions for preventing disagreeable post-operative conditions were systematically taken, surgery would have a better reputation and patients would submit to its ministrations with less reluctance. He has some ideas of his own that he realizes will not meet general approval but his experience teaches him not to use scissors. SHARP knives only are needed. Haemostats are applied too frequently and make wounds painful. Silk sutures should be used rather than the absorbable ones that must be digested by the tissues and thus cause local hyperaemias wherever they are placed. As few incisions as possible should be made and there should be no "piddling." Secondary operations are largely due to adhesions and these can be avoided by the use of silk, in his opinion.

Dr. L. C. Childs said that a mouth mask would prevent some infection. One had only to note the salivary ejections in conversations to be satisfied that they also occurred at the operating table when surgeons had to speak to assistants.

Dr. E. L. Griffin said that the patient should have all the rest possible both before and after operation. Purging should be done two days in advance of work. The preparation of the field should be wide and complete and the incision large enough for internal palpation. He believes in it because it has disclosed things that he was totally unable to discover by the best diagnostic means. It is done successfully at the Mayo clinics. It takes nerve sometimes but it often prevents secondary operations.

Time is saved by good instruments kept in good condition. The surgeon should assure himself of his instrument before each sitting. The instruments should be boiled; no other method of sterilization is safe in his opinion. Post-operatively he uses morphine, high enemas and pituitrin as indicated; stomach lavage has no value as a rule.

Dr. E. D. Highsmith said that examination and diagnosis as aided by team work with laboratory men and X-Ray observations are of the utmost and prime importance. In the work, itself, permanent assistants are of great value both in saving time and in doing things well. We can not all have permanent anaesthetists under the present conditions. Besides, men who are giving anaesthetics are likely to be interested in the operations so that they may become proficient in operative work themselves. For this reason, he looks with favor upon the lady anaesthetist. She has won her place and pays attention to her work alone.

Dr. W. A. Selman said that during the years when he had been giving anaesthetics, he watched the operators closely and learned from them things he could not have acquired in any other way. The patient need not be neglected on this account. It is commendable for the man to know all the conditions he may have to meet.

Dr. G. D. Ayer expressed the opinion that anaesthesia requires great skill and that only a carefully trained man should give it. The nurse who has studied it specially for a few weeks has a limited knowledge only.

Dr. M. T. Davis protested against the giving of morphine for even a few days to relieve post-operative pains. He instances cases of men who were born inebriates, but who had never had the opportunity to use the drug, having becomes habitues after operation.

He spoke against careless and rough handling of the wounds as well as against Trendelenberg position.

In closing Dr. Westmoreland said that nine-tenths of the infection came from the skin of the patient. Th proof of this lies in the absence of infection when the skin is cleansed and iodine is used. As to gloves, if the preparation of the hands as McRae stated, showed no bacteria even after a long operation, why use gloves? Westmoreland doesn't use them himself, and he has as little infection as anyone. He prepares the patient's skin several hours in advance and keeps wet antiseptic dressing upon it if possible. It takes 200,000 bacteria to cause an infection and well cleansed hands will not carry that many any more than the ordinary air will infect a wound. The Crile anoci association has called for careful dissection in surgery, but this may be speedily done by the well equipped man. Gauze packing always means traumatism and reduces vitality. Cell resistance is reduced by even saline douches and there should be nothing touching the wound except the blood. A dry field is essential. As to sutures, the question is, will they do what you want them to perform? He can not find in town any gut that is guaranteed to last as long as the label says it will, and the circulars he has received from suture manufacturers tell him of things he can not secure. There is no standardized gut to be had. The giving way of gut causes infections and adhesions. Silk and linen have their disadvantages but they do hold. As to stitching he believes in close work by many sutures and he has no post-operative hernias as a result. He has had to perform many secondary operations on this account because of insufficient suture support in the first instance. Vomiting is less likely to occur in an empty stomach than a full one, and he keeps food away from his patients until they are ready for it. As to thirst, he saturates them with water in advance and doesn't try to control the mouth thirst except with a little lemon juice.

Dr. McRae said that large incisions and internal palpation have caused indiscriminate surgery and increased morbidity. If we diagnose in advance as we should, we don't need the exploration. If he were a patient, himself, he would want the surgeon to know in advance just what was to be done. He does not believe in blunt dissection nor does he approve of laying scissors aside. Good sharp instruments and care in rapid dissection give him his results. He might be accused of "piddling" because he does tie every vessel and covers every surface and uses sutures enough to close every wound tightly.

As to wearing gloves, the hands do contain germs in the skin that can not be eliminated entirely. These come out with the sweat and will infect a wound after a time. As to sutures, he believes that it is better to digest an absorbable one than to incapsulate a permanent one. Anaesthesia demands a qualified man and the laws do not permit others to do this part of the practice of medicine. In Louisiana the courts have decided against it. No one who has not taken a full course of study in reaching a medical degree even begins to be competent to give anaesthetics.

Dr. Nicolson said that for severe vomiting he has long been giving small doses of apomorphia with excellent results. He doesn't know why but it stands the pragmatic test. If it should not work on the second dose, it will not be of use at all. He believes in giving small doses of morphine after operating because of the great comfort they furnish and if some few men get the habit because of idiosyncrasy, they might better have it than to let all the patients suffer for the want of it. He doesn't use gloves and can't perceive the need of them. He wants to keep the "eyes in his fingers" unblinded.

Dr. E. G. Jones said that in his experience there is nothing to be done about the nausea and vomiting except a masterly inactivity. As Dr. Collier had said a patient who came to the table with a strongly acid urine and all that it stood for, there would be marked nausea. If this were relieved in advance, the comfort of the patient would be increased. A surgeon should protect a patient in every possible way and having accustomed assistants is one of the best ways. During the last decade many men have been recommending getting patients out of bed very early. He doesn't agree with them. Rest is necessary to the restoration of the continuity of tissues and ten days is not a bit too long for them to remain quiet in bed.

Dr. J. S. Derr showed X-Ray plates of a chronic appendix displaying residue after 24, 53 and 72 hours and another after 6 days. It is a poorly drained appendix and required operation for relief. Another patient was skiagraphed because of lack of relief after appendix had been removed. The plate showed marked heptic kink and several adhesions. Acute appendicitis can not be diagnosed by the X-Ray.

